# Avian Neuropeptide Y: Beyond Feed Intake Regulation

**DOI:** 10.3390/vetsci9040171

**Published:** 2022-04-01

**Authors:** Elizabeth S. Greene, Nedra Abdelli, Jalila S. Dridi, Sami Dridi

**Affiliations:** 1 Department of Poultry Science, University of Arkansas, Fayetteville, AR 72701, USA; esgreene@uark.edu (E.S.G.); nedra.abdelli@uab.cat (N.A.); 2 Animal Nutrition and Welfare Service, Department of Animal and Food Sciences, Universitat Autònoma de Barcelona, 08193 Bellaterra, Spain; 3 École Universitaire de Kinésithérapie, Université d’Orléans, Rue de Chartres, 45100 Orleans, France; jaliladr2@gmail.com

**Keywords:** neuropeptide Y, feed intake regulation, adipose tissue, liver, immune system, gut, muscle, bone, chickens

## Abstract

Neuropeptide Y (NPY) is one of the most abundant and ubiquitously expressed neuropeptides in both the central and peripheral nervous systems, and its regulatory effects on feed intake and appetite- have been extensively studied in a wide variety of animals, including mammalian and non-mammalian species. Indeed, NPY has been shown to be involved in the regulation of feed intake and energy homeostasis by exerting stimulatory effects on appetite and feeding behavior in several species including chickens, rabbits, rats and mouse. More recent studies have shown that this neuropeptide and its receptors are expressed in various peripheral tissues, including the thyroid, heart, spleen, adrenal glands, white adipose tissue, muscle and bone. Although well researched centrally, studies investigating the distribution and function of peripherally expressed NPY in avian (non-mammalian vertebrates) species are very limited. Thus, peripherally expressed NPY merits more consideration and further in-depth exploration to fully elucidate its functions, especially in non-mammalian species. The aim of the current review is to provide an integrated synopsis of both centrally and peripherally expressed NPY, with a special focus on the distribution and function of the latter.

## 1. Introduction

Neuropeptide Y (NPY) is a 36-amino acid peptide, which along with peptide YY (PYY) and pancreatic polypeptide (PP), belongs to the pancreatic polypeptide family. First isolated from pig brain in 1982 [1], NPY is considered to be the most conserved peptide among vertebrate species. It is one of the most abundant and ubiquitously expressed neuropeptides in both the central and peripheral nervous systems [2], with the arcuate nucleus (also known as the infundibular nucleus in birds) as its main source [3,4]. Considered as a potent orexigenic neuropeptide, NPY is one of the major regulators of feed intake, energy homeostasis and appetite in several species. Indeed, central injection of NPY has been shown to increase food intake in rats [5,6], sheep [7], pigs [8], and chickens [9,10]. Moreover, feed deprivation in rodents has been shown to up-regulate the hypothalamic expression of NPY to incite feed intake and maintain homeostasis [11], while rats subjected to repeated administration of NPY show hyperphagia and increased body weight gain leading to obesity [12]. Aside from its role in the stimulation of food intake, NPY has been also shown to be expressed in peripheral tissues but its functions are still not fully elucidated. NPY has been shown to be highly expressed in the adrenal glands [13], white adipose tissue [14], and bone [15,16] among others, indicating the potential involvement of this neuropeptide in a wide range of physiological responses including adipogenesis, regulation of bone mass, and energy metabolism [17], locomotion [18], anxiety [19] learning and memory [20], epilepsy [21], circadian rhythm [22], and cardiovascular function [23]. To exert its effects, NPY binds to specific NPY receptors, which are members of the class A G-protein coupled receptors [24].

Chicken is considered as an important scientific and commercial species and the focus on studying the growth and metabolism of chicken, both of which are influenced by NPY, led to the identification of avian NPY in the early 1990s [25]. Recently, six receptor subtypes for NPY have been identified and cloned in diverse avian tissues (Y1, Y2, Y4, Y5, Y6, and Y7) [26,27,28] and their characteristics have been investigated.

Although central administration of NPY seems to have a similar effect on feed intake regulation [29] in mammalian and avian species [30], much effort is still needed to understand the physiological functions of peripheral NPY in chickens. Therefore, the purpose of the current paper is to review the physiological functions of avian NPY at both the central and peripheral level, for subsequent identification of research gaps that need to be addressed in the future.

## 2. Structure of NPY

**Figure 1:**
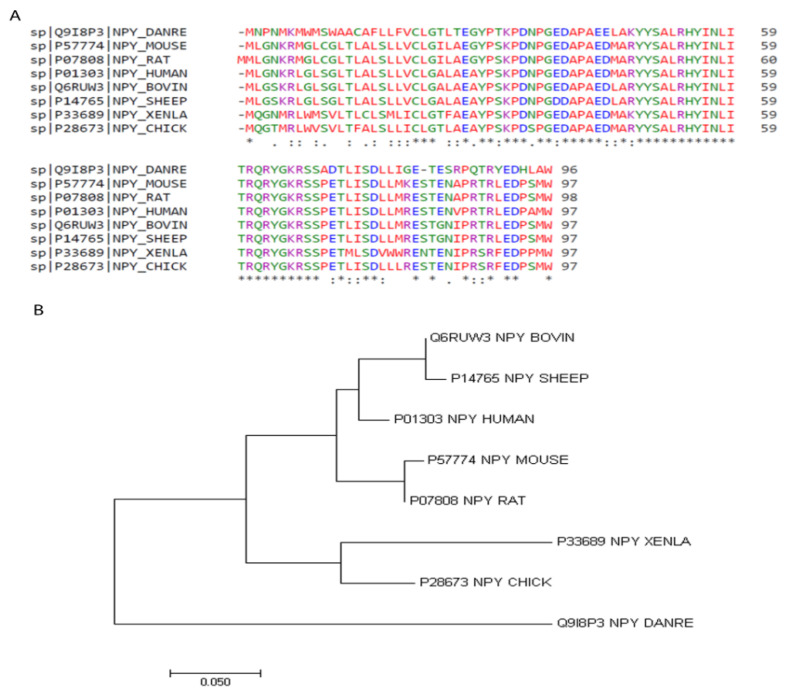
NPY amino acid sequence alignments (**A**) and phylogeny (**B**). Amino acid sequences were aligned using Clustal Omega 1.2.4 [32]. * positions with a single, fully conserved residue. “:” (colon) conservation between groups of strongly similar properties. “.” (period) conservation between groups of weakly similar properties. Phylogenetic tree generated with MEGA7: Molecular Evolutionary Genetics Analysis version 7.0 for bigger datasets [33].

The NPY system is an ancient signaling pathway, as it is found in both vertebrates and invertebrates, highlighting a potential evolutionarily conserved function [31]. Structurally similar to PYY and PP, the amino acid sequence of NPY is one of the most highly conserved neuropeptides. As shown in Table 1, there is over 90% identity in the amino acid sequence among mammalian species, and greater than 80% identity between chicken and other species (Figure 1A) [32]. Additionally, phylogenic analysis indicates that the non-mammalian species share a common ancestor that diverged from mammals in their NPY sequence [33] (Figure 1B). The molecular structure contains numerous hydrophobic interactions, as well as and N-terminal polyproline-II-like helix and a C-terminal α-helix [34]. The N-terminal portion is responsible for interactions with various receptors, as studies have shown this segment interacts with Y1 but not Y2 [35,36]. Additionally, NPY contains two translation initiation sequences, allowing for the production of both full-length and a truncated NPY, containing only peptides 17–36 [37], which can further differentially bind to receptors.

## 3. NPY Receptors

The physiological effects of NPY are exerted through binding to specific Y receptors, which are part of the G-protein-coupled (GPCR) family [24]. To date, 7–8 different receptors have been identified, though their presence and functionality differs among species. In mammals, Y1, Y2, Y4, Y5, and Y6 have been identified [38], whereas in fish, chicken and other avian species, Y7 is additionally present [27], and in frogs [39] and telost fish [40], Y8a and Y8b may also be present. The Y receptors have a long evolutionary history and are grouped into three subfamilies based on the homology and similarity of their amino acid sequences. The Y1 subfamily consists of Y1, Y4, Y6, and Y8, with sequence homology ranging from 40 to 60% [41]. Y1 only binds to intact NPY and PYY peptides [42,43], whereas Y4 preferentially binds PP over NPY or PYY [44]. Additionally, Y4 shows low sequence homology among different species, making it one of the most rapidly evolving GPCR known [41]. Interestingly, Y6 is the most variable in expression and functionality across species, with a complete absence in rat [45], it is truncated in many other mammals or in specific tissues [46], and present and functional in chicken [27,28,47]. The Y2 family consists of Y2 and Y7, and likely arose from a gene duplication of Y1 in an invertebrate ancestor, creating Y2 [31]. Unlike Y1, Y2 can bind truncated forms of NPY in mammals and chicken [48,49], though with less affinity in fish [50]. The Y5 subfamily consists of a single member, Y5, that similarly came from a duplication of Y1, after the creation of Y2 [31], but only has approximately 20% sequence homology with Y1 or Y2 [51]. Along with Y1, Y5 is the receptor responsible for the canonical orexigenic effects of NPY [52,53,54,55].

## 4. NPY Downstream Signaling Cascades

**Figure 2:**
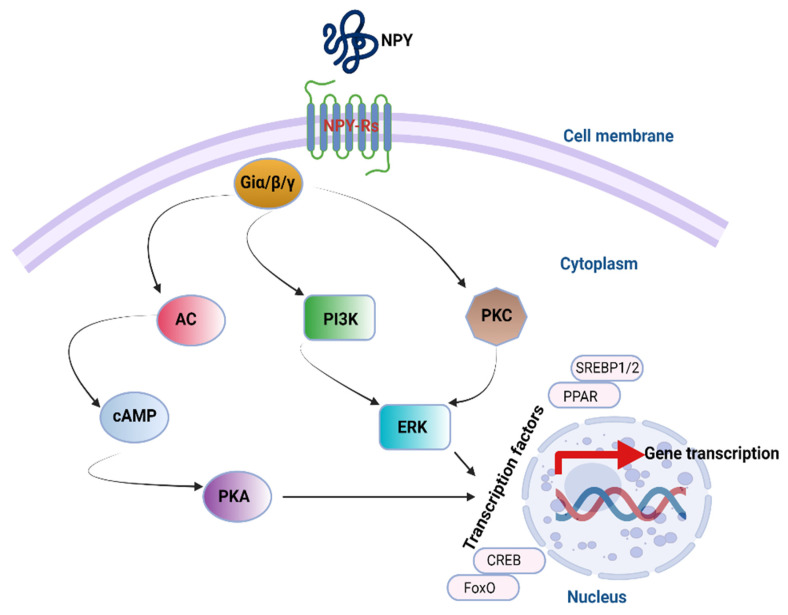
NPY downstream signaling pathways. The representation shows the potential main pathways through which NPY signals. AC, adenylate cyclase; cAMP, cyclic adenosine monophosphate; CREB, cAMP response element binding protein; ERK, extracellular signal-regulated kinase; FoxO, Forkhead Box O; PI3K, phosphatidylinositol-3-kinase; PKA, protein kinase A; PKC, protein kinase C; PPAR, peroxisome proliferator-activated receptor; SREBP, sterol regulatory element-binding protein. The figure was made using Biorender.com.

Mammalian NPY receptors couple primarily through Giα to inactivate adenylate cyclase (AC) and decrease cAMP synthesis, which in turn leads to a reduction in protein kinase A (PKA) activity [56]. NPY receptors can also, through Gq or Giβ/γ, activate phospholipase C (PLC) and protein kinase C (PKC), which in turn induces mitogen-activated protein kinase (MAPK) activation including the phosphorylation of the extracellular signal-regulated kinase (ERK1/2) [57]. A phosphatidylinositol-3-kinase (PI-3-K) pathway upstream of ERK1/2 activation has also been identified [58] (Figure 2). Although such downstream signaling is not well confirmed in chickens, phosphorylated levels of protein kinase b (Akt), forkhead box protein O1 (FOXO1), and ribosomal protein S6 kinase (S6K) were increased in the hypothalamus of fasted and re-fed chicks, which correlated with increases in the plasma concentration of insulin [59]. Similarly, central administration of insulin by ICV injection increased the phosphorylation of Akt, FOXO1, and S6K in the hypothalamus of chicken. Central inhibition of PI3K (by LY294002) or mTOR (by rapamycin) was able to increase the feed intake, further highlighting possible NPY involvement [59]. However, further studies are warranted to fully determine whether or not these downstream mediators are activated by NPY.

## 5. Tissue Distribution of NPY System

NPY was first discovered in the porcine brain [1], and has since been well characterized in the brain and central nervous system of numerous species [60,61,62,63,64], with the greatest concentration seen in the arcuate nucleus of the hypothalamus [65]. In the chicken, NPY is widely distributed across the brain [66], with in situ hybridization studies identifying high abundances of NPY mRNA in specific neurons, including the hippocampus, nucleus commissurae pallii, infundibular hypothalamic nucleus, nucleus pretectalis pars ventralis and neurons around the nucleus rotundus [67].

Outside of the central nervous system, NPY and its receptors have more recently been identified in the periphery, though its role in these tissues is still being elucidated. Bone [15], adipose tissue [14], platelets [68] and intestine are all known mammalian sources of NPY. In chicken, NPY has also been identified in numerous tissues, including ovary, testes, heart, kidney, lung, skeletal muscle, fat, pancreas, liver, and intestine [28,47,69], though it has not been detected in spleen. The Y1 receptor is expressed in all chicken tissues studied to date, though at differing relative abundance, with greater expression in the ovary, heart, kidney, liver, and muscle [28,47]. The Y2 receptor has similar tissue expression in chicken as in rainbow trout [50] and frog [39], with the heart, duodenum, liver, lung, muscle, ovary, testes, pituitary, spleen, and pancreas [26] all expressing this receptor. Interestingly, this differs from humans, where Y2 is often lowly detected or undetectable [70]. These differences likely relate to some of the different effects seen in mammalian and non-mammalian species, such as NPY-mediated lipid accumulation in chickens [71]. Likely due to their similarity in structure [27], Y7 shows a comparable pattern of wide tissue distribution, and likely serves an overlapping functional role as well. There have been few studies examining Y5 in the periphery. However, gene transcripts have been amplified in pancreas, testes, ovary, duodenum [26], and muscle [47] of chicken.

## 6. Physiological Functions of NPY

### 6.1. Central Functions of NPY

**Figure 3:**
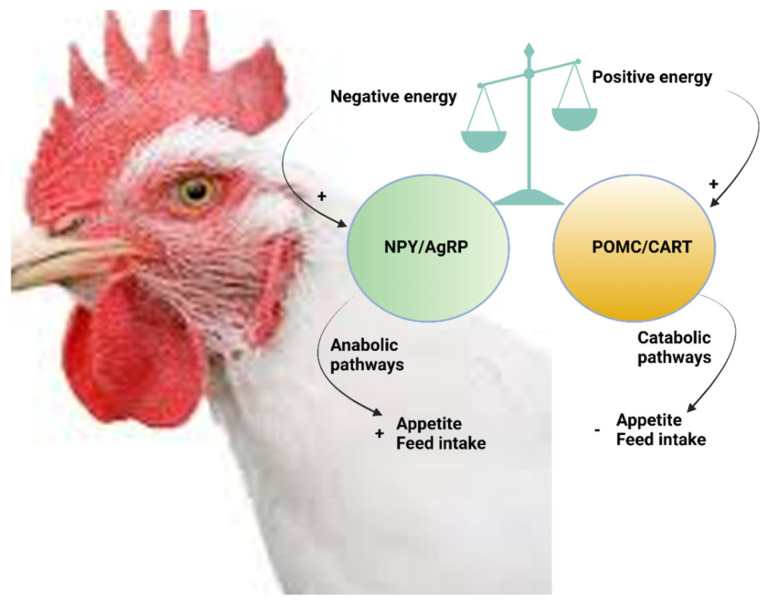
A proposed model describing central feed intake regulation in chickens through hypothalamic (an)orexigenic neuropeptides. AgRP, agouti-related peptide; NPY, neuropeptide Y; POMC, proopiomelanocortin. (-) inhibition; (+) stimulation. The figure was made using Biorender.com.

The balance between feed intake and energy homeostasis is a complex system of regulatory mechanisms and pathways in multiple organ systems. These mechanisms include signaling molecules such as nutrients, metabolites, hormones, neuropeptides, and receptors that originate from the central and peripheral nervous system, as well as other tissues such as the gut, muscle, liver, and adipose. Together, these molecules interact via feedback mechanisms to convey signals and information about the whole-body nutrient status of an organism [72,73]. In the arcuate nucleus (the equivalent of the infundibular nucleus in chickens) of the hypothalamus, feeding signals are integrated by two groups of neurons with opposing functions [74]. Stimulation of the NPY/agouti-related protein (AgRP) neurons results in an orexigenic response and increased energy intake and storage, whereas stimulation of the proopiomelanocortin (POMC) and cocaine/amphetamine-regulated transcript (CART) neurons induces a decrease in energy intake and storage [72] (Figure 3). Indeed, intraperitoneal injection of recombinant NPY increased feed intake in broiler (meat-type) chickens [47], being in concordance with previous studies using central administration of NPY in broilers [30,75,76,77] and layer (egg-type) chickens [78,79]. The effects of NPY on feeding behavior of chickens are mediated by Y1 and Y5 [80]. On the contrary, the encoded precursor protein of POMC in chickens was shown to produce bioactive alpha-melanocyte stimulating hormone (αMSH). The injection of this hormone has been shown to inhibit feed intake in chickens [77], an effect antagonized by AgRP through binding and signaling to specific melanocortin receptor subtypes (MC3R and MC4R) [72]. On the other hand, previous studies have shown NPY mRNA levels to be up-regulated in the hypothalamus by fasting [81], feed restriction [82], and down-regulated by insulin [83] and leptin [84].

### 6.2. Peripheral Functions of NPY

#### 6.2.1. NPY in Adipose Tissue

The factors that regulate energy balance are complex and are not limited to the central nervous system. Energy storage, particularly in adipose tissue, the primary energy reservoir for the body, is an important component of the overall energy status of an organism. White adipose tissue contains a heterogeneous mixture of mature adipocytes, preadipocytes, mesenchymal stem cells, immune cells, and a matrix of collagen fibers, and is the main adipose depot for energy storage in the form of triacylglycerol. Brown adipose, on the other hand, is responsible for heat production and non-shivering thermogenesis; however, this form of fat has not been found in chicken [85]. Concurrently, with the known role of NPY in centrally regulating feed intake and energy balance, it is not surprising that it also plays a role in cross-talk between the hypothalamus and adipose tissue. Several studies have shown that the sympathetic nerve terminals in adipose tissue, as well as adipocytes themselves [86], can secrete NPY and promote adipogenesis and inhibit lipolysis [87].

**Figure 4:**
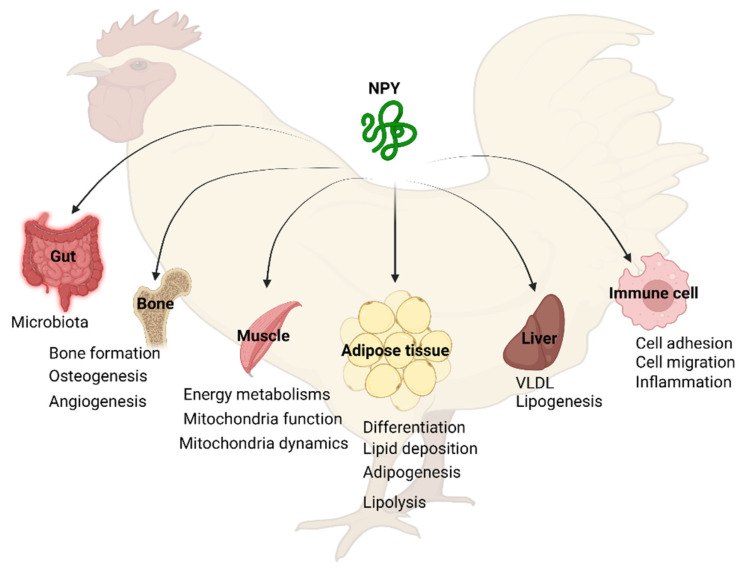
Peripheral physiological functions of NPY. VLDL, very low-density lipoprotein. The figure was made using Biorender.com.

Much of our understanding of avian NPY in adipose comes from in vitro studies using culture of pre-adipocytes isolated from the abdominal fat of chickens. In these cells, treatment with NPY promotes differentiation and lipid accumulation, an effect mediated by Y2 [71]. During differentiation, the addition of NPY is associated with increased glycerol-3-phosphate dehydrogenase (GAPDH) activity, which leads to the production of glycerol-3-phosphate, a key molecule in triacylglycerol synthesis. Additionally, gene expression of several important transcription factors involved in adipocyte differentiation (peroxisome proliferator-activated receptor gamma (PPARγ), CCAAT/enhancer binding protein alpha (C/EBPα), and sterol regulatory element-binding protein 1 (SREBP1)), are all affected by NPY [71] (Figure 4). Similarly, NPY treatment promotes preadipocyte activity during the early phase of chick development. This results in increased lipid accumulation, enhanced expression of SREBP, C/EBPβ, and fatty acid binding protein 4 (FABP4), increased GAPDH activity, and decreased expression of Krüppel-like factor 7 (KLF7) and DNA topoisomerase II alpha (TOP2A), all indicative of greater adipogenic activity [88].

These results have more recently been verified in vivo, as NPY, Y1, and lipolytic factors adipose triglyceride lipase (ATGL) and FABP4 were decreased in the subcutaneous fat depot from day 0 to 4 post-hatch, indicating the involvement of the NPY system in the mobilization of fat from this early-life energy reservoir [89].

NPY has also been shown to inhibit lipolysis via Y1. This was evidenced through the reduction in plasma non-esterified fatty acids (NEFAs) at 1 and 12 h post injection of NPY in chickens [90]. On the other hand, peripheral NPY has been shown to differentially affect adipogenesis and lipolysis in chicks from lines selected for low (LWS) or high body weight (HWS) [90]. The results of this study indicated higher rates of lipolysis in LWS and adipogenesis in HWS.

#### 6.2.2. NPY in the Liver

The regulation of hepatic lipid homeostasis is at least partially controlled by interaction with the central nervous system, and defects within this interaction have been associated with dyslipidemia, such as obesity, diabetes, and metabolic syndrome [91]. As such, nerve fibers within the liver have been shown to express NPY in mammalian (mouse, rat, guinea pig, dog, monkey, and human) and non-mammalian (carp, bullfrog, turtle, and chicken) species [92]. In mammalian studies, centrally produced NPY has been shown to be involved in lipid [91,93,94] and glucose [94,95] metabolism regulation, both biological processes regulated by the liver. Central administration of NPY increased very low-density lipoprotein (VLDL) secretion in rats, independently of food intake [93]. This effect is mediated by Y1 as the Y1 agonist, [F7, P34]-NPY, increased VLDL secretion via activation of stearoyl-CoA desaturase 1 (SCD1), ADP-ribosylation factor 1 (ARF1), and lipin1, all necessary factors for VLDL maturation and secretion [91]. More recently, it has been shown that hepatic stellate cells can secrete NPY and that NPY is important in the fibrogenic response that can be seen in diseased liver states [96].

In chicken, NPY and its receptors are expressed in the liver [69,97], except for Y7 [27], though their role has yet to be fully explored. NPY and Y1, Y2, and Y5 all increase in hepatic expression from day 4 to 14 post-hatch [98], a time during which lipogenesis is increasing. Unlike most mammals, where the liver and adipose share the role of de novo lipogenesis, in chicken over 95% of de novo fatty acids are synthesized by the liver [99], making this a key organ in overall energy homeostasis. Therefore, further research into the role of hepatic NPY in chicken may impact not only the poultry industry, but has potential effects as a clinically relevant human model for lipid dysmetabolism.

#### 6.2.3. NPY in the Muscle

The modern chicken has been selected for fast growth rate and feed efficiency [100], with the majority of this increase seen in the commercially important breast muscle [101]. As NPY has a significant role in energy balance regulation, it is conceivable that it could be an important mediator of muscle growth. Indeed, several recent studies have characterized the expression of the NPY system in avian muscle and myoblast cells. Similar to the effect seen centrally, fasting increased the gene expression of NPY and its receptors in both breast and leg muscle of 9-day-old broilers [47]. In the same study, intraperitoneal administration of NPY up-regulated gene expression of the NPY system in leg and breast muscle, particularly Y1, Y2, Y4, Y6, and Y7, though this effect was dose dependent in leg muscle, with increases seen at the low dose and decreases at the higher doses [47]. This suggests that NPY regulates its own system and that NPY might have paracrine/autocrine functions. Of particular interest, NPY was also able to regulate mitochondrial function in this metabolically active, fast-growing tissue, indicating that NPY plays a crucial role in muscle energy metabolism.

Satellite cells are multi-potent cells that are important in muscle fiber growth and regeneration. These cells fuse with existing muscle fibers and donate their nuclei, thereby increasing protein production and hypertrophy, and allowing for muscle growth [102]. Studies using isolated chicken [103] and turkey [103,104] satellite cells have shown that NPY is expressed by these cells and can be regulated by environmental factors. The effects of thermal manipulation on the expression of the NPY system seem to be dependent on the bird lines from which the cells were isolated, as well as the state of the cells (proliferative vs. differentiating). As modern commercial broilers and turkeys have a relatively narrow range of thermotolerance and are thereby susceptible to heat stress [105], these changes in NPY and NPY receptors suggests that, in muscle, this neuropeptide may act as a hypothermic regulatory agent. In chicken-derived satellite cells, the response to incubation temperature was dependent on the type of birds from which the cells were isolated. In proliferating cells isolated from two lines of chicken, neither NPY nor its receptors were greatly affected by temperature. However, NPY, Y2, and Y5 were increased by elevated temperature in differentiating satellite cells from Ross 708 broilers [103]. Similarly, NPY expression was increased in turkey-derived satellite cells when incubated at 41 [104] or 43 °C [103] as compared to lower temperatures. Concurrently, expression of receptors Y2 and Y5 were increased in proliferating satellite cells, whereas only Y2 was affected by increased temperature in differentiating satellite cells [103].

#### 6.2.4. NPY in the Bone

More than just a structural support for the body, bone is a dynamic tissue that undergoes remodeling throughout the life of an organism. Bone homeostasis balances resorption and formation, which when in disequilibrium, can lead to changes in the microarchitecture and integrity of bone tissue [106]. The bone is innervated [107], giving the potential for a direct link with centrally-mediated processes. Indeed, the NPY system has more recently been identified as one of the key regulators of this important process as it is secreted by nerve fibers in the marrow and vascular canals, and the Y1 [15,108] and Y2 [109] receptors have been implicated in bone homeostasis. The regulation of bone mass via NPY differs at the hypothalamic and bone level. Osteoblast specifc-Y1 knockout studies in mice have shown that this receptor is critical for the actions of NPY directly at the bone [108], whereas Y2 is critical for the central regulation of bone mass [110]. In further support of these distinct roles, the expression of Y1 has been reported in osteoblastic bone marrow-derived mesenchymal stem/stromal cells (BMSCs) and osteocytes, whereas Y2 is yet undetected in bone cells. Interestingly, the effects of NPY in vivo are different from that seen in isolated BMSCs. In vivo, during fasting when hypothalamic NPY is high, bone formation is reduced [16]. However, when studies are conducted in vitro, particularly with BMSCs, the results can be controversial [111]. For instance, some studies showed an inhibition of BMSC proliferation and osteoblast differentiation by NPY [112], while others reported enhanced BMSC proliferation and osteoblastic activity with NPY treatment [113,114,115]. Regardless, the effects of NPY seem to be conferred through the Wnt signaling pathway [111]. This pathway is activated by NPY in a dose-dependent manner, where downstream activation of β-catenin and phospho- glycogen synthase kinase-3 beta (p-GSK-3β) occurs, as well as an up-regulation of the osteoblastic genes alkaline phosphatase (ALP), collagen type I, osteocalcin and Runx2 [111]. The role of NPY in chicken and other avian species has yet to be explored. Given the importance of bone disorders, such as bacterial chondronecrosis with osteomyelitis (BCO), as well as the importance of bone health and metabolism to egg production [116] in the poultry industry, this provides an open avenue for future research.

#### 6.2.5. NPY in Macrophage and Immune System

Innervation of immune organs constitutes one of the primary ways in which NPY regulates immune function [117]. Additionally, though basal levels are low, upon stimulation or immune activation, immune cells can also directly produce NPY and up-regulate NPY receptors, leading to autocrine and paracrine effects [118]. The Y1 receptor is present in almost every type of immune cell, including lymphocytes, natural killer cells, dendritic cells, granulocytes, and monocytes/macrophages [119]. Early studies in mice showed that NPY can modulate the immune response by acting as a chemical attractant, decreasing adhesion and promoting migration and phagocytosis of peritoneal macrophages [120,121]. These effects are mediated by the Y1 receptor; however, under different physiological or pathological conditions, activation of the Y2 receptor can increase adhesion [122] and decrease migration of monocytes and leukocytes [123]. The differences in downstream effects upon receptor binding are related to dipeptidyl peptidase 4 activity, which specifically terminates NPY-Y1 interactions and also changes with age [122,124]. These differences highlight the complexity of the NPY response and the importance of NPY-receptor interactions. Based on the known differences in sequence homology of these receptors among species, it is quite likely that the effects of NPY in chicken immune cells differ, or exert effects through different receptors, compared to mammalian species.

NPY also exerts inflammo-modulatory effects through cytokine production. These effects can be either pro- or anti-inflammatory, depending on the cell type and mode of activation. For instance, activated RAW246.7 macrophages showed increased expression of tumor necrosis factor alpha (TNFα), C-reactive protein (CRP), and monocyte chemoattractant protein 1 (MCP1), all of which were decreased by co-incubation with an Y1 antagonist [125]. In addition, in isolated mouse macrophages and human whole blood from healthy subjects, NPY increases interleukins (IL-1b, IL-6) and TNFα [126,127]. However, NPY produced by adipose tissue macrophages inhibits the expression of IL-6 and TNFα through the autocrine and paracrine systems [128].

Finally, NPY has also been shown to indirectly regulate immune function through pathways that affect obesity, diabetes, mood, and thermoregulation, all of which can then modulate the immune response. Of particular importance in chicken is the interaction between NPY and heat stress. Indeed, NPY has been shown to induce hypothermia in birds [129,130], and is known to be modulated by heat stress in birds [131]. As this state also induces inflammation, further study of the role of NPY and its interaction with the immune system during this critical physiological state may provide future insights into helping the poultry industry manage heat stress.

#### 6.2.6. NPY in the Gut

With feed intake controlled by the hypothalamus and nutrients absorbed by the gastrointestinal tract, the term “gut–brain axis” refers to the critical and complex communication that controls energy homeostasis. The system is bidirectional, in that signals from the brain regulate motility, secretion, digestion, absorption in the gut, and the gut sends signals relating to nutrient and energy status back to the central nervous system. NPY is present at all levels of this axis, and within the gut, it is primarily expressed by the enteric neurons [132]. Because of this, NPY is able to regulate a wide range of functions of the intestine, including motility and epithelial permeability, as well as the immune-related functions of cytokine production and inflammation [133]. In mammals, centrally administered NPY delays gastric emptying, likely via interaction with Y2 receptors, as determined by receptor-inhibition studies [134,135]. Additionally, NPY has an anti-secretory and pro-absorptive effect [136,137], particularly in the retention of chloride ions [133,138]. In Caco2 cells, it has been shown that NPY exerts this effect by increasing the association between the Cl¯/HCO3¯(OH¯) transporter (SLC26A3) and membrane lipid rafts [139]. To date, most of the gastric effects of NPY have been attributed to its interactions with Y1 or Y2; however, multiple NPY receptor subtypes are present within the intestine, suggesting that the variety of functions of NPY may result from this diversity, though the exact interactions and consequences are yet to be elucidated. Y4, in particular, is present in both the mammalian and chicken [28,97] gut, though it may mediate the effects of PP over NPY [133].

As the importance of the microbiota in whole-body health has become recognized in recent years, it is not surprising that neuropeptides also play a role in the gut microbiome. NPY has been shown to have anti-microbial properties, with the ability to inhibit the growth of *E. coli* in vitro [140]; however, studies with other organisms such as *S. aureus* and *C. albicans* have shown conflicting results [140,141,142]. The mechanism behind this potential inhibition seems to come from both direct disruption and depolarization of the cell membrane [143,144,145], and indirectly via modulation of intestinal inflammation [146]. This effect has yet to be studied in avian species, but does present an interesting and potentially impactful area for future research.

## 7. Regulation of Avian NPY Expression

The regulation of avian NPY expression involves nutritional, hormonal, genetic, and environmental factors. Indeed, early studies showed that negative energy conditions such as food restriction and deprivation enhance hypothalamic NPY mRNA expression [82] and neuron activity [147]. A study conducted by Zhou et al. [148] showed that chickens subjected to fasting for up to 72 h exhibited increased NPY content in the hypothalamic infundibular nucleus (IN) and paraventricular nucleus (PVN), but not in the lateral hypothalamic area (LHA). In the PVN, NPY returned to pre-fasting levels after 24 h of re-feeding. However, the level of NPY was unaffected in the IN, suggesting that fasting and re-feeding of broiler chickens can differentially affect NPY in the brain. A more recent study showed an increased NPY expression associated with lowered feed intake, particularly in 3-week-old cockerels, confirming that NPY is associated with the nutritional state of chickens [148]. Additionally, gene expression of NPY and other orexigenic molecules were up-regulated in low growth rate as compared to high growth rate cockerels, corroborating the findings reported by previous studies [149,150]. Moreover, the increase in NPY was associated with an overexpression of brain-specific homeobox protein (BSX). This confirms the requirement of BSX for physiological expression of NPY/AgRP and stimuli of hyperphagic response in avian species as demonstrated in mice [151].

In long-term divergently selected chickens, for the ratio of abdominal fatness to live weight, Dridi’s group found that the hypothalamic expression of NPY was higher in fat compared to lean bird lines under both fed and fasted conditions [152]. The same group found that the hypothalamic expression of NPY was lower in high- compared to low-feed efficient male quails, but it remained unchanged between female lines [153], indicating a potential gender-dependent effects.

NPY expression is also regulated by hormonal factors such as insulin, leptin, and glucocorticoids (GCs). These peripheral hormonal signals are integrated in the hypothalamus at the arcuate nucleus of mammals or infundibular nucleus of birds [154,155]. Intracerebroventricular (ICV) injection of GCs increases feed intake in chicks [156] in a dose-dependent manner, whereas infusion of recombinant leptin over a 6 h period significantly reduced feed intake in 3-week-old broiler chickens. This effect was mediated via selective down-regulation of the hypothalamic expression of NPY [157].

Moreover, a study was conducted with the aim to evaluate the effect of dietary energy level and feeding state on the GC-induced gene expression of hypothalamic feeding-related neuropeptides, including NPY [158]. The results showed that dexamethasone treatment significantly increased hypothalamic NPY expression under fasting conditions. This effect was observed in chickens fed a high-fat diet but not in their counterparts receiving a low-fat diet, suggesting that the effect of peripheral GCs injection on NPY expression is dependent on dietary energy concentration. The same study showed a decrease in hypothalamic NPY levels under re-feeding conditions.

ICV injection of insulin has been shown to inhibit feed intake in young chickens via the central melanocortin system [83]. Similarly, ICV injection of insulin had an anorexgenic effect on leghorn and broiler chicks [159,160], indicating that insulin in birds, like mammals, is an anorexigenic neuropeptide. More recent studies have further explored the interaction between NPY and insulin, and have indicated that the hypophagic effect of insulin is likely mediated by the Y1 and Y2 receptors [161,162].

Environmental conditions may also alter NPY expression; however, the data are controversial and a matter of debate. For instance, heat-stressed chickens showed an increased NPY mRNA [163,164], decreased NPY mRNA [125], or no change when compared to controls [165], though these effects may differ based on age and strain of the birds studied, as well as the temperature and duration of the heat stress. Similarly, ICV injection of NPY during heat exposure diminished the orexigenic response of broiler chicks to NPY [131], while heat-stressed layer-type chicks, ICV-injected with NPY responded similarly to thermoneutral chicks [129]. Moreover, NPY treatment has been shown to exert a hypothermic effect on layer-type chickens [129,166], however this has yet to be explored in broilers, but does suggest that NPY additionally inhibits energy expenditure. The reduction in NPY abundance and function observed in some studies is a plausible explanation for the decrease in food intake during heat stress, whereas the increase reported by other authors may be induced by the increase in plasma corticosterone under stressful conditions.

## 8. Conclusions and Perspectives

In summary, avian NPY plays a key role in feed intake regulation, consistent with the results obtained in mammals. Several studies demonstrated that this neuropeptide is also expressed in various peripheral tissues, suggesting a pleotropic physiological functions. However, much effort is still required to determine the exact physiological functions and their associated downstream mechanisms in such tissues.

## Figures and Tables

**Table 1:** Amino acid sequence homology of NPY among several species.

	Zebrafish	Mouse	Rat	Human	Bovine	Sheep	Xenopus	Chicken
Zebrafish	100.00	67.71	67.71	66.67	65.62	64.58	62.50	65.62
Mouse	67.71	100.00	98.97	92.78	90.72	89.69	74.23	81.44
Rat	67.71	98.97	100.00	93.81	91.75	90.72	75.26	82.47
Human	66.67	92.78	93.81	100.00	94.85	93.81	78.35	84.54
Bovine	65.62	90.72	91.75	94.85	100.00	98.97	76.29	84.54
Sheep	64.58	89.69	90.72	93.81	98.97	100.00	75.26	83.51
Xenopus	62.50	74.23	75.26	78.35	76.29	75.26	100.00	84.54
Chicken	65.62	81.44	82.47	84.54	84.54	83.51	84.54	100.00

Numbers indicate percent identity between species, as determined by Clustal Omega 1.2.4 [26].

## Data Availability

All the data are included in the review.
